# Transcatheter epicardial pacing of the human Bachmann’s bundle via the transverse sinus: Novel technique to expand transcatheter cardiac intervention

**DOI:** 10.1016/j.hrcr.2026.06.027

**Published:** 2026-06-30

**Authors:** Yuichiro Miyazaki, Shumpei Mori, Mark Rimmer, Venkatakrishna Tholakanahalli

**Affiliations:** 1 David Geffen School of Medicine at the University of California, Los Angeles, California; 2 Cardiology Division, University of Minnesota, Minneapolis, Minnesota; 3 Cardiology Division, Minneapolis VA Health Care System, Minneapolis, Minnesota

**Keywords:** Bachmann’s bundle, Epicardial pacing, Interatrial synchrony, Transverse sinus, Atrial synchronization

**Key Teaching Points**

• Transcatheter epicardial mapping of Bachmann’s bundle via the transverse sinus is technically feasible.
• Transcatheter epicardial pacing of Bachmann’s bundle accelerates left atrial activation and improves left atrioventricular synchrony.
• Percutaneous transcatheter access to the transverse sinus has immense potential to expand the field of cardiac intervention and device implantation.

## Introduction

As atrial lead implantation in the right atrial appendage is technically straightforward, the right atrial appendage remains one of the common sites for atrial pacing. However, it induces interatrial conduction delay, resulting in interruption of the left atrial wave by mitral valve closure, which prevents effective left ventricular filling.^1^, ^2^, ^3^, ^4^ Interatrial dyssynchrony can be appreciated by the prolongation of the mean P-wave duration during right atrial appendage pacing (132 ms) compared with that during sinus rhythm (108 ms).^1^^,^^5^^,^^6^ In addition, active fixation into the right atrial appendage is not without risk of perforation.^7^ Alternative pacing approaches include atrial septal pacing and Bachmann’s bundle pacing. Mean P-wave duration during atrial septal pacing (95 ms) and Bachmann’s bundle pacing (93 ms) is shorter than that during sinus rhythm and right atrial appendage pacing.^1^^,^^3^ Although both approaches prevent interatrial dyssynchrony,^3^^,^^4^ Bachmann’s bundle pacing can also attenuate the progression of atrial fibrillation with stable capture thresholds during the follow-up period.^1^^,^^2^^,^^8^, ^9^, ^10^, ^11^, ^12^, ^13^, ^14^ Thus, Bachmann’s bundle pacing is now greatly preferred as the better alternative to conventional right atrial appendage pacing.^15^ This trend of revisiting Bachmann’s bundle pacing is evident in the rapid increase in publications on “Bachmann’s bundle pacing” since 2024, following the first trend around 2002 in PubMed searches. This trend has also been facilitated by recent industrial improvements, or vice versa, including the development of the thin-caliber lumenless active fixation lead and 3-dimensionally pre-shaped guiding catheter. However, as Bachmann’s bundle is an epicardial interatrial connection, selective pacing of Bachmann’s bundle is only feasible from the epicardial aspect during cardiac surgery via the transverse sinus.^16^^,^^17^ To the best of our knowledge, epicardial pacing of Bachmann’s bundle has never been performed with a less-invasive transcatheter epicardial approach. As opposed to a classic endocardial attempt,^9^ we herein report a case of transcatheter epicardial Bachmann’s bundle pacing via the transverse sinus to guide subsequent endocardial Bachmann’s bundle pacing.

## Case report

**Figure 1:**
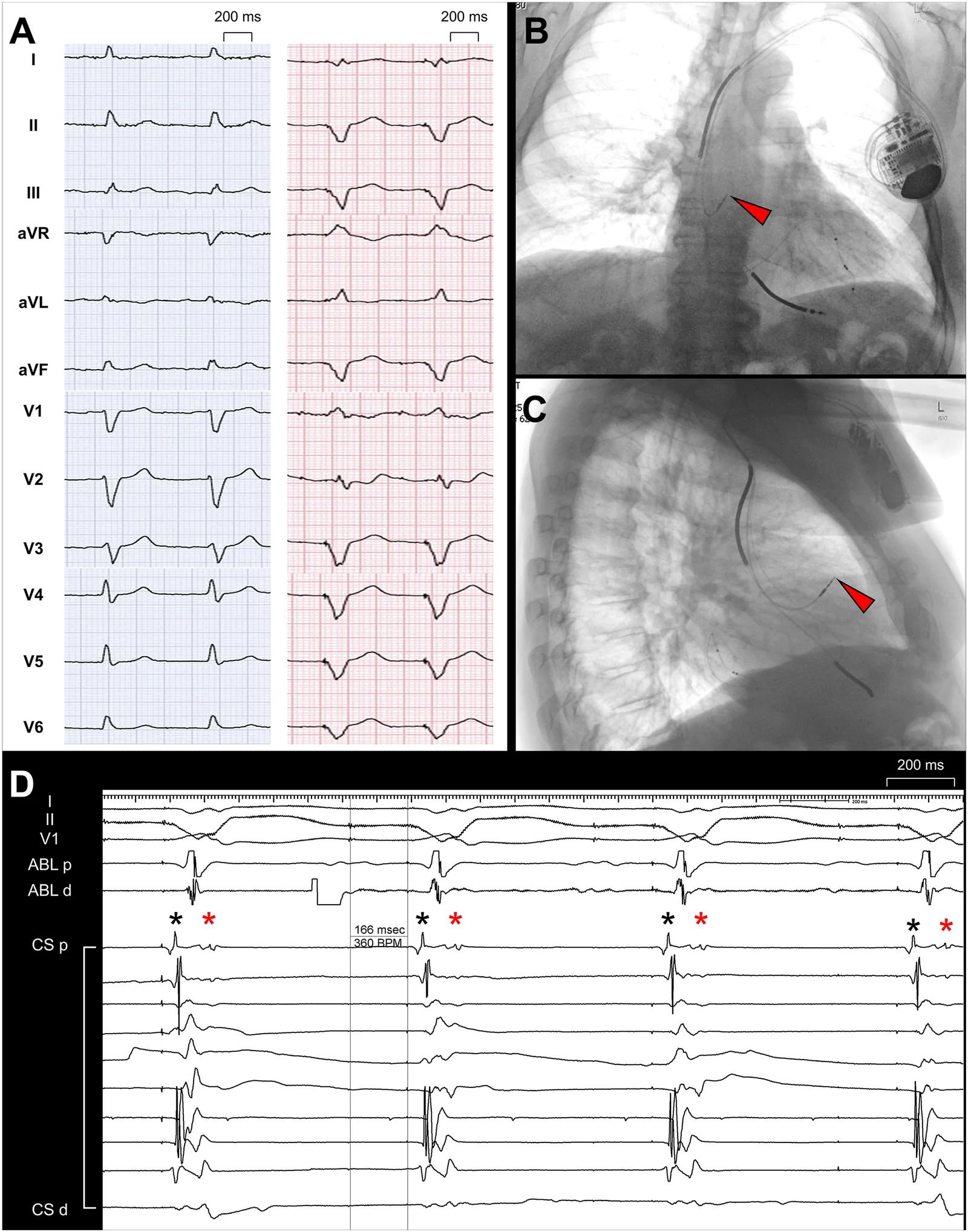
Baseline electrocardiograms, chest radiographs, and electrogram. 12-lead electrocardiogram during sinus rhythm (**A**, *left*) exhibits low amplitude in the P wave. P-wave duration measured from lead aVL was 113 milliseconds. During right atrial appendage and biventricular pacing (**A**, *right*), P waves are hardly detected, and thus, P-wave duration could not be measured. The frontal (**B**) and lateral (**C**) chest radiographs show the atrial lead implanted within the right atrial appendage (*arrowheads*). The intracardiac electrogram (**D**) during right atrial appendage and biventricular pacing (atrioventricular [AV] delay of 170 milliseconds, adaptive cardiac resynchronization therapy mode) revealed simultaneous left atrial (*black asterisks*) and ventricular (*red asterisks*) signals within the QRS wave, as recorded in the coronary sinus (CS). ABL = ablation catheter; CS = coronary sinus catheter; d = distal; p = proximal.

A 69-year-old male patient presented for catheter ablation for drug-resistant ventricular tachycardia. He had a history of congestive heart failure (New York Heart Association class III), non-ischemic cardiomyopathy, hypertension, dyslipidemia, diabetes mellitus, chronic kidney disease, and prostatic cancer. He had also undergone cardiac resynchronization therapy (CRT) with defibrillator implantation (Claria MRI™ Quad CRT-D SureScan™; Medtronic, Minneapolis, MN), programmed in DDDR mode, at 60/105 pulses per minute, and active adaptive CRT mode with a right atrial appendage lead. His medications included amiodarone (400 mg/day) and ranolazine (1000 mg/day). He had no history of atrial fibrillation and was free from anticoagulation. The electrocardiogram recorded during sinus rhythm before CRT exhibited a low-amplitude P wave. However, after CRT, the P wave could not be detected (Figure 1). Transthoracic echocardiography depicted a dilated left ventricle (left ventricular end-diastolic dimension, 61 mm) with reduced left ventricular ejection fraction (20%). Both atria were also dilated, and transmitral pulsed-wave Doppler recording revealed E-wave and A-wave velocity as 1.05 m/s and 0.03 m/s, respectively.

Baseline intracardiac electrogram (EGM) during right atrial appendage and biventricular pacing demonstrated simultaneous left atrial and ventricular signals, as recorded in the coronary sinus. This observation of “pacemaker syndrome”^18^ indicated a significant delay in the left atrial excitation and resultant loss of interatrial and atrioventricular (AV) synchrony (Figure 1). This is not hemodynamically optimal for patients with severe left ventricular dysfunction and significantly reduced atrial booster function, as evidenced by low-voltage P waves during sinus rhythm.

**Figure 2:**
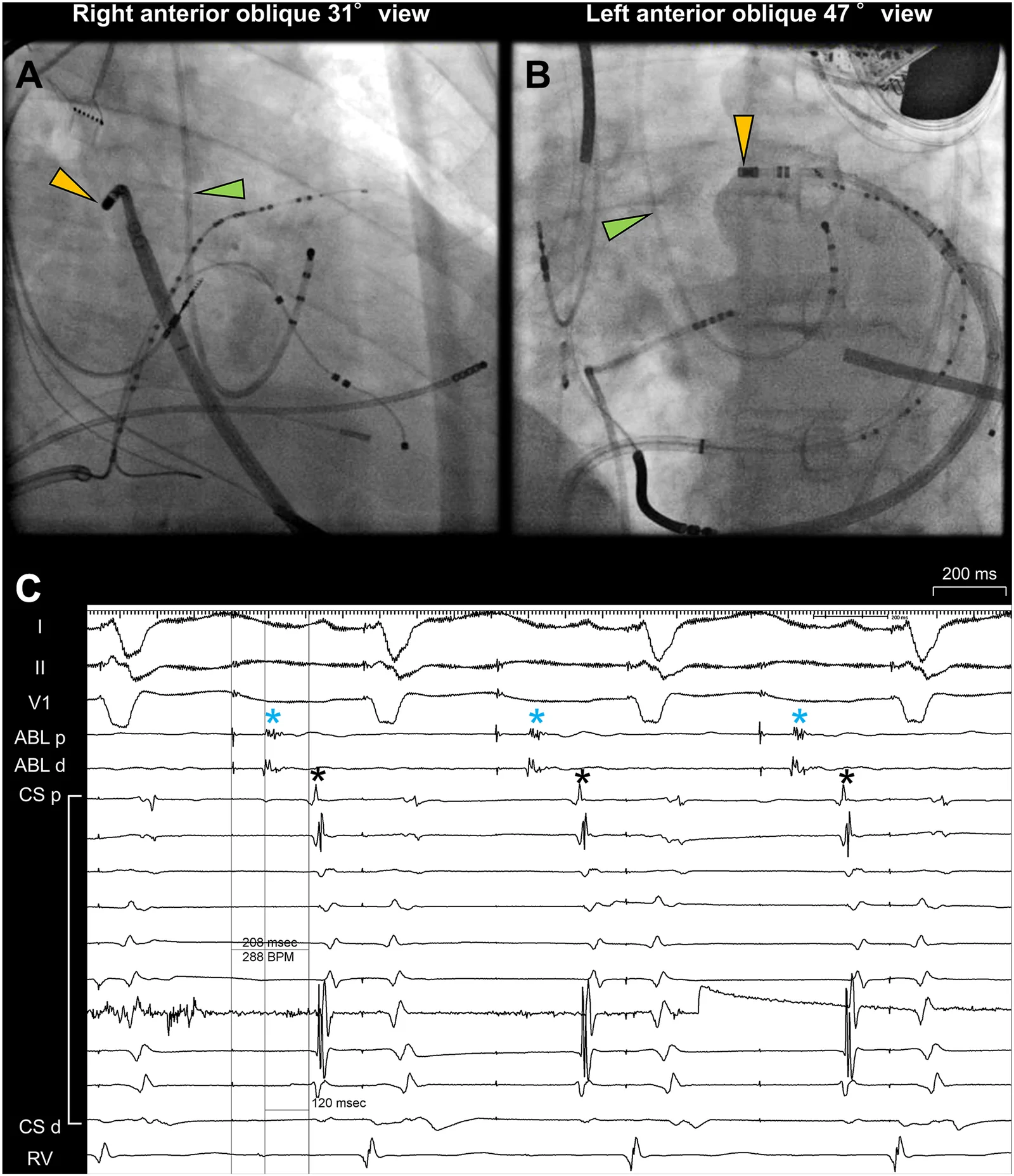
Fluoroscopic image and intracardiac electrocardiogram of the ablation catheter in the transverse sinus. The tip of the ablation catheter (*orange arrowheads*) is located posterior to the catheter (*green arrowheads*) placed in the ascending aorta in the right anterior oblique view (**A**). With the support of a deflectable guiding sheath, the ablation catheter, via the inferior epicardial approach, enters the transverse sinus from its left-side entrance and reaches the medial border of the vertebral column, but still lateral to the catheter placed in the ascending aorta and far lateral to the leads in the superior vena cava in the left anterior oblique view (**B**). Intracardiac electrogram recorded by the ablation catheter during right atrial appendage and biventricular pacing (with prolonged AV delay of 360 milliseconds) displays separation of atrial signals (*black asterisks*) from the QRS wave and fractionated Bachmann’s bundle signal (*blue asterisks*) 88 milliseconds after the pacing spike. The left atrial signal in the proximal CS electrode is recorded 208 milliseconds after the atrial pacing spike, also delayed by 120 milliseconds from the onset and by 55 milliseconds from the endpoint of Bachmann’s bundle potential (**C**), representing the interatrial conduction delay. ABL = ablation catheter; AV = atrioventricular; CS = coronary sinus; d = distal; p = proximal; RV = right ventricle.

At first, catheter ablation of ventricular tachycardia associated with the extensive basal left ventricular scar was performed using both endocardial and epicardial access via the inferior approach. Substrate modification rendered clinical ventricular tachycardia non-inducible. Subsequently, we attempted to guide the epicardial ablation catheter (THERMOCOOL SMARTTOUCH™ SF; Johnson & Johnson MedTech, New Brunswick, NJ) into the transversus sinus with the support of a guiding sheath (Agilis™ EPI Steerable Introducer; Abbott, Abbott Park, IL) (Figure 2). The attempt was straightforward, and we could insert the catheter from the left-side entrance to the transverse sinus and place it on the area we interpreted as Bachmann’s bundle based on the biplane fluoroscopic images and a fractionated signal (Figure 2). The paced AV delay was prolonged to allow separation of atrial and ventricular signals, with atrial signals preceding the QRS (Figure 2). A clear P wave was observed in lead I, and the P-wave duration estimated from the EGM was at least >200 milliseconds (>120 milliseconds is deemed abnormal). In this prolonged-paced AV delay setting, QRS morphology changed due to intrinsic right ventricular excitation-gated biventricular pacing activated by the ventricular sense response program (Figure 2).

**Figure 3:**
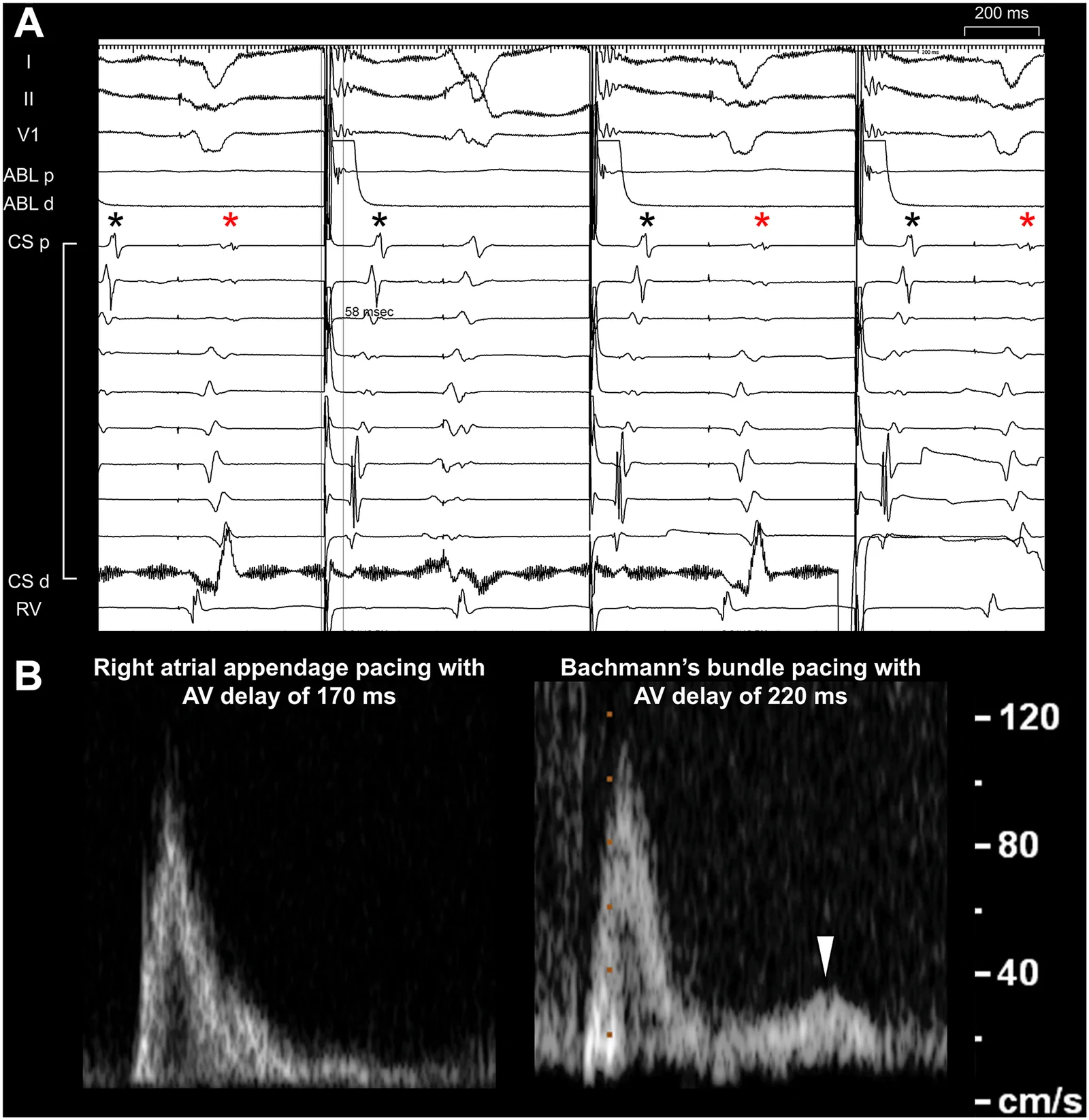
Intracardiac electrogram during Bachmann’s bundle pacing from the epicardial side using the ablation catheter. Bachmann’s bundle was paced from the epicardial side through the transversus sinus using the ablation catheter (**A**), which revealed shortening of the conduction time from pacing to the left atrium (from 208 milliseconds to 58 milliseconds). By the shortening of this interval and prolonged AV delay, optimal separation between the left atrial signals (*black asterisks*) and ventricular signals (*red asterisks*) was confirmed, preventing AV dyssynchrony. All beats except the second beat (premature ventricular contraction from the left ventricular summit) are intrinsic right ventricular excitation-gated biventricular pacing activated by the ventricular sense response program. The sequence of atrial signals recorded by the CS catheter also changed from Chevron-shaped (Figure 2) to distal-to-proximal propagation. A wave in the transmitral flow (*arrowhead*) is restored after the procedure without truncation (**B**). ABL = ablation catheter; AV = atrioventricular; d = distal; p = proximal; RV = right ventricle.

Direct transcatheter epicardial pacing of Bachmann’s bundle was feasible. Optimal separation between the left atrial signals and ventricular signals was confirmed (Figure 3). However, during Bachmann’s bundle pacing, the interval between the atrial pacing spike and the right atrial activation was not discerned, as the right atrial catheter was not placed.

**Figure 4:**
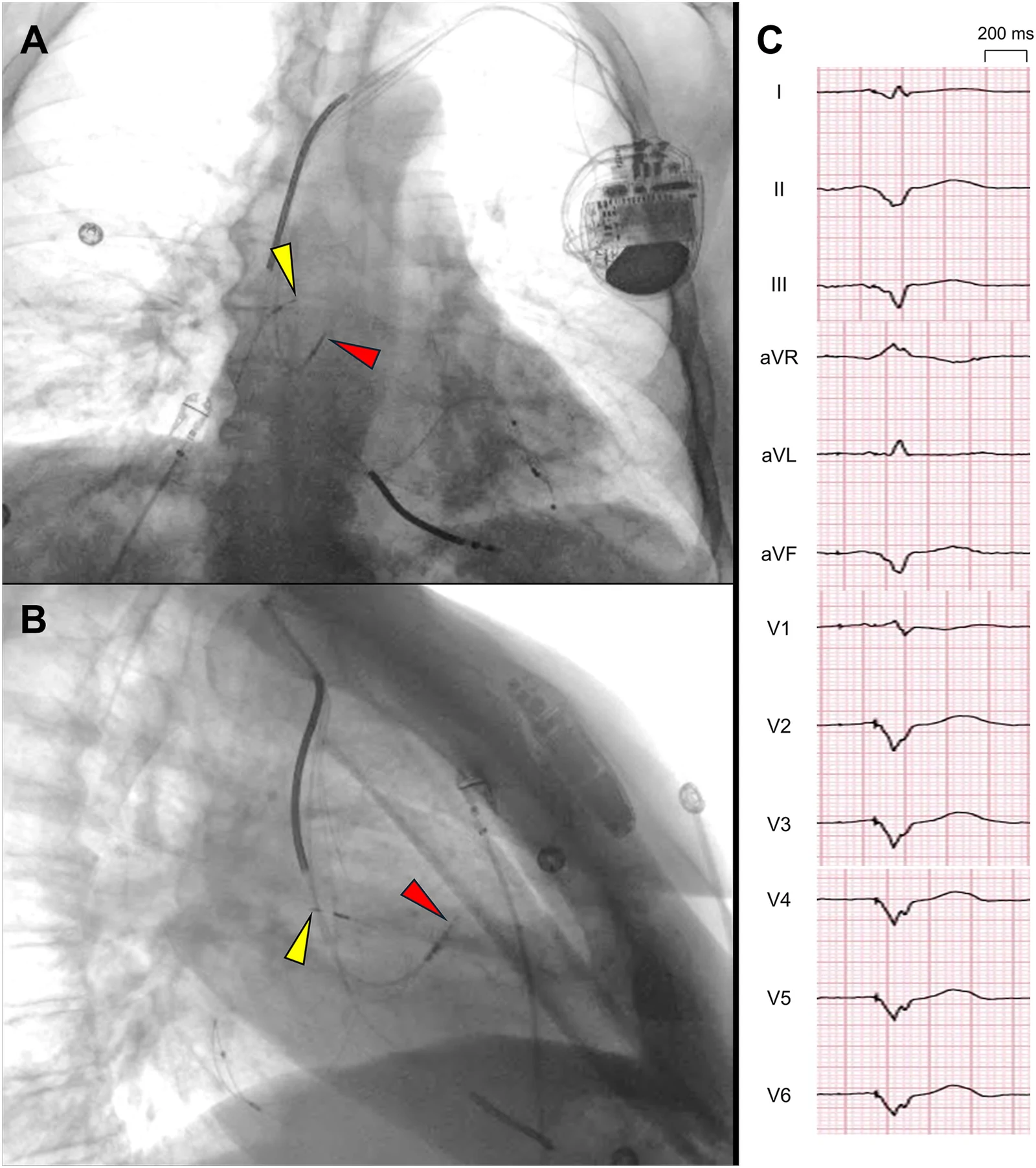
Frontal and lateral radiographs and electrocardiogram of Bachmann’s bundle pacing lead implantation. Bachmann’s bundle pacing lead (*yellow arrowheads*) directs towards the posteromedial direction, whereas the old right atrial appendage pacing lead (*red arrowheads*) directs towards the anteromedial direction (**A, B**). P wave detection during endocardial Bachmann’s bundle pacing remains challenging from the standard electrogram (**C**).

**Figure 5:**
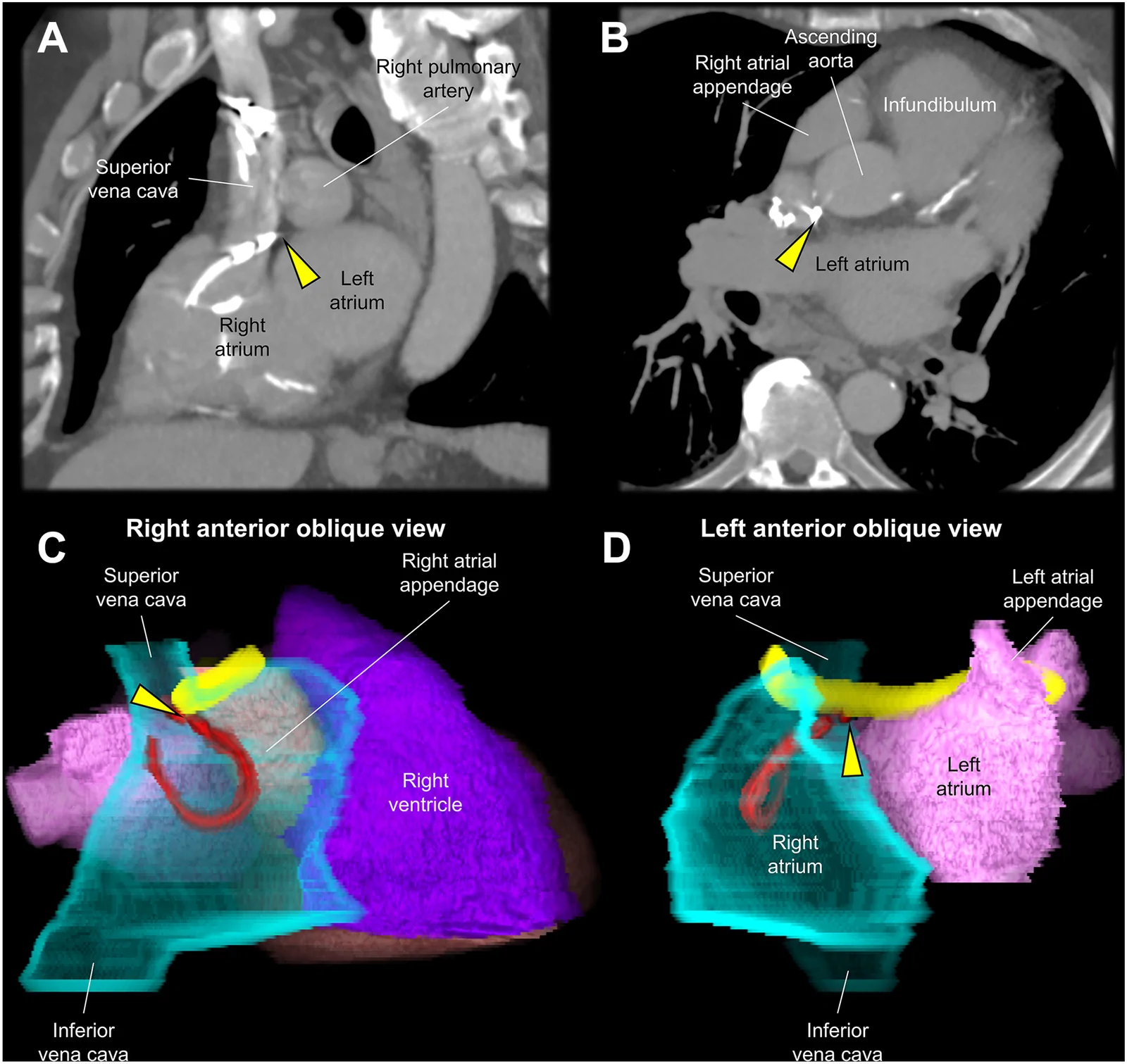
Contrast-enhanced thoracic computed tomography after Bachmann’s bundle pacing lead implantation. Multiplanar reconstruction images viewed from the inferior (**A**) and left anterior oblique (**B**) views depict the tip of the lead placed at the posteromedial area of the atriocaval junction adjacent to the interatrial portion of Bachmann’s bundle covering the anterosuperior interatrial groove. Volume-rendered images viewed from the right (**C**) and left (**D**) anterior oblique directions, reconstructed with virtual transverse sinus (*yellow*) and Bachmann’s pacing lead (*red*), depict the relationship between the lead and surrounding structures relative to the transverse sinus.

Based on these findings from the acute study that preferred Bachmann’s bundle pacing over the right atrial appendage pacing in addition to AV delay optimization, we decided to schedule endocardial Bachmann’s bundle pacing. 3 months after catheter ablation, Bachmann’s bundle pacing was performed targeting the posteromedial area of the atriocaval junction (Figures 4 and 5). Furthermore, during implantation, the coronary sinus catheter was used to monitor the conduction time from pacing to the left atrium. The conduction time from pacing to the coronary sinus improved from 225 milliseconds to 135 milliseconds when compared to right atrial appendage pacing. Pacing threshold was 1.25 V at 0.4 milliseconds with an impedance of 1083 Ω, and the amplitude of the atrial signal was 1.6 mV. The new atrial lead (SelectSecure™ MRI SureScan™ 3830 Lead; Medtronic) was connected to the CRT device, and the old atrial lead was capped. The postprocedural CRT device was set to DDDR mode at 60–120 pulses per minute, with a paced AV delay of 220 milliseconds and a sensed AV delay of 200 milliseconds. AV delay was optimized based on transmitral flow pattern and velocity time integral at the left ventricular outflow tract measured by transthoracic pulsed-wave Doppler echocardiography (Figure 3). After the ablation and CRT upgrade with Bachmann’s bundle pacing, his New York Heart Association class improved from class III to class II without hospitalization due to heart failure. Although P-wave duration remains challenging to measure, follow-up transthoracic echocardiography revealed improved left ventricular ejection fraction (26%) with negative remodeling (left ventricular end-diastolic dimension, 55 mm). Optimal AV synchrony was confirmed by restoration of appropriate atrial booster function in transmitral flow pattern without truncation (E-wave velocity 1.09 m/s, A-wave velocity 0.29 m/s) (Figure 3). However, the patient required bilateral cardiac sympathetic denervation because of recurrent ventricular tachycardia.

**Figure 6:**
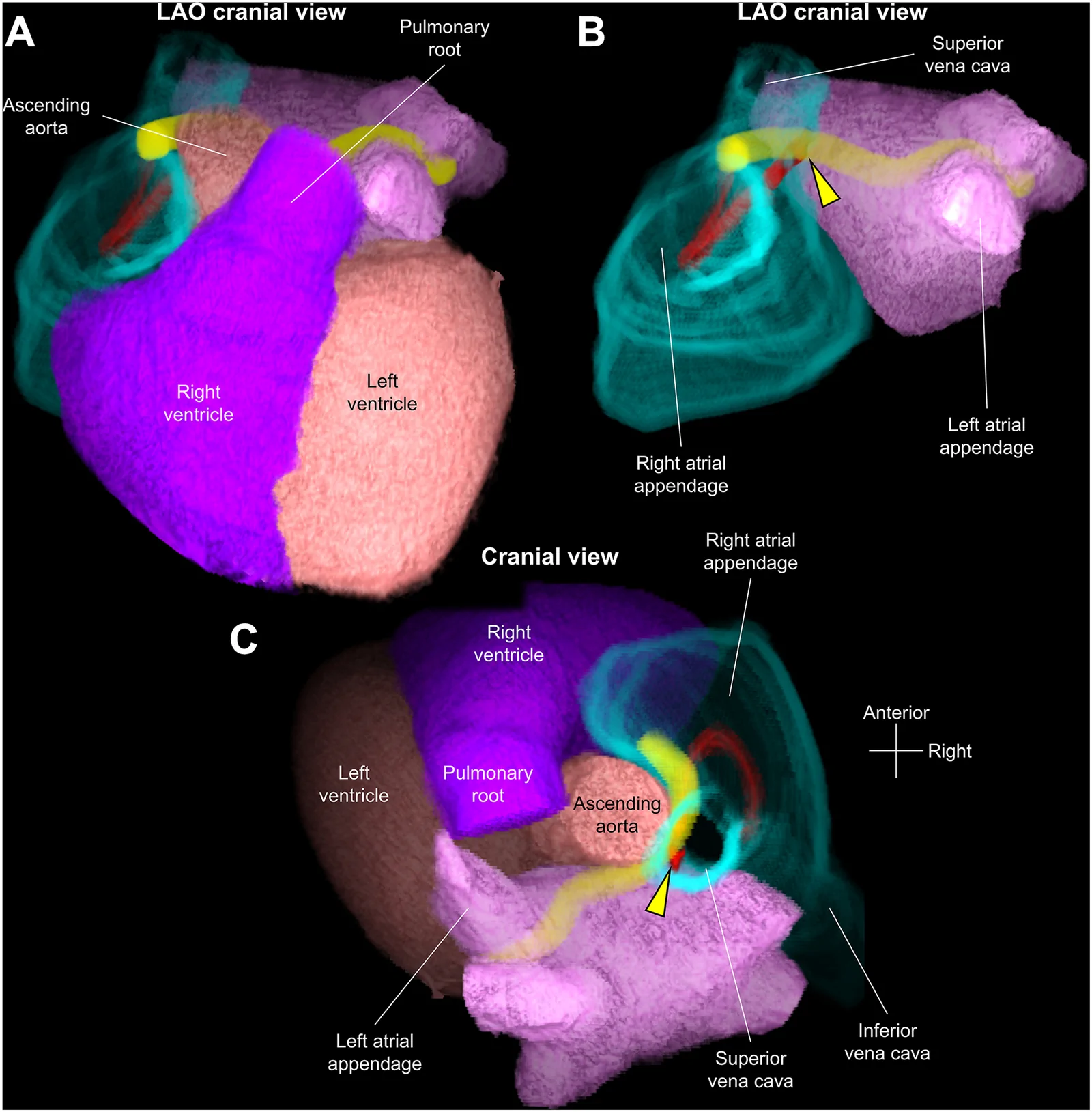
Clinical anatomy of the transverse sinus relevant to Bachmann’s bundle pacing. Volume-rendered images are reconstructed with the virtual transverse sinus (*yellow*) and Bachmann’s bundle pacing lead (*red*) and are viewed from the left anterior oblique (**A, B**) and superior (**C**) directions. As the transverse sinus is located along the right anterior to left posterior direction (**C**), its en-face view can be obtained from the left anterior oblique direction (Figure 5) with (**A, B**) or without cranial angulation (Figure 5). LAO = left anterior oblique.

**Figure 7:**
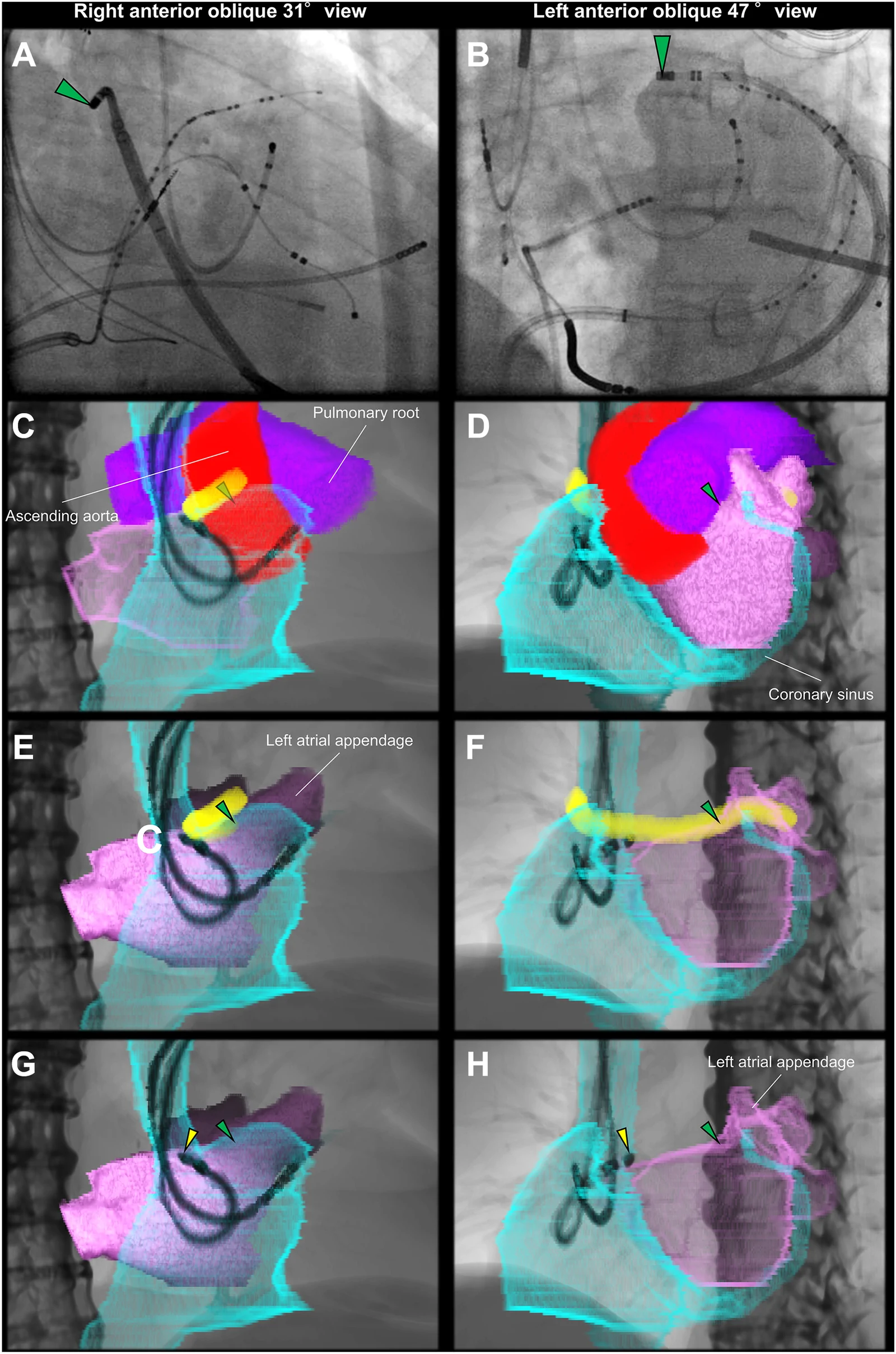
Review of the transcatheter epicardial pacing site of Bachmann’s bundle. By using identical right (**A**) and left (**B**) anterior oblique directions applied during transcatheter epicardial Bachmann’s bundle pacing, volume-rendered images (**C–H**) are reconstructed with the virtual transverse sinus (*yellow*), right atrial appendage lead, and Bachmann’s bundle pacing lead. Such a 3-dimensional comparison can clarify the site of transcatheter epicardial pacing (*green arrowheads*) via the transverse sinus using its left-side entrance. It is located on the anterior wall of the left atrium just medial to the left atrial appendage orifice, before passing under the pulmonary trunk. That location is 39.8 mm lateral to the endocardial Bachmann’s bundle pacing site (*yellow arrowheads*).

Contrast-enhanced thoracic computed tomography obtained after the procedure confirmed optimal anatomical location of Bachmann’s bundle pacing lead in association with the transverse sinus (Figures 5 and 6). Furthermore, by detailed 3-dimensional comparison with biplane fluoroscopic images (Figure 2), the site of transcatheter epicardial Bachmann’s bundle pacing via the transverse sinus was confirmed to be the left-side extension of the bundle located on the anterior wall of the left atrium (Figure 7).

## Discussion

The main message of this case report is that transcatheter epicardial mapping of Bachmann’s bundle via the transverse sinus is technically feasible without complication and also useful to guide the subsequent endocardial Bachmann’s bundle pacing.

### Anatomy and physiology of Bachmann’s bundle

**Figure 8:**
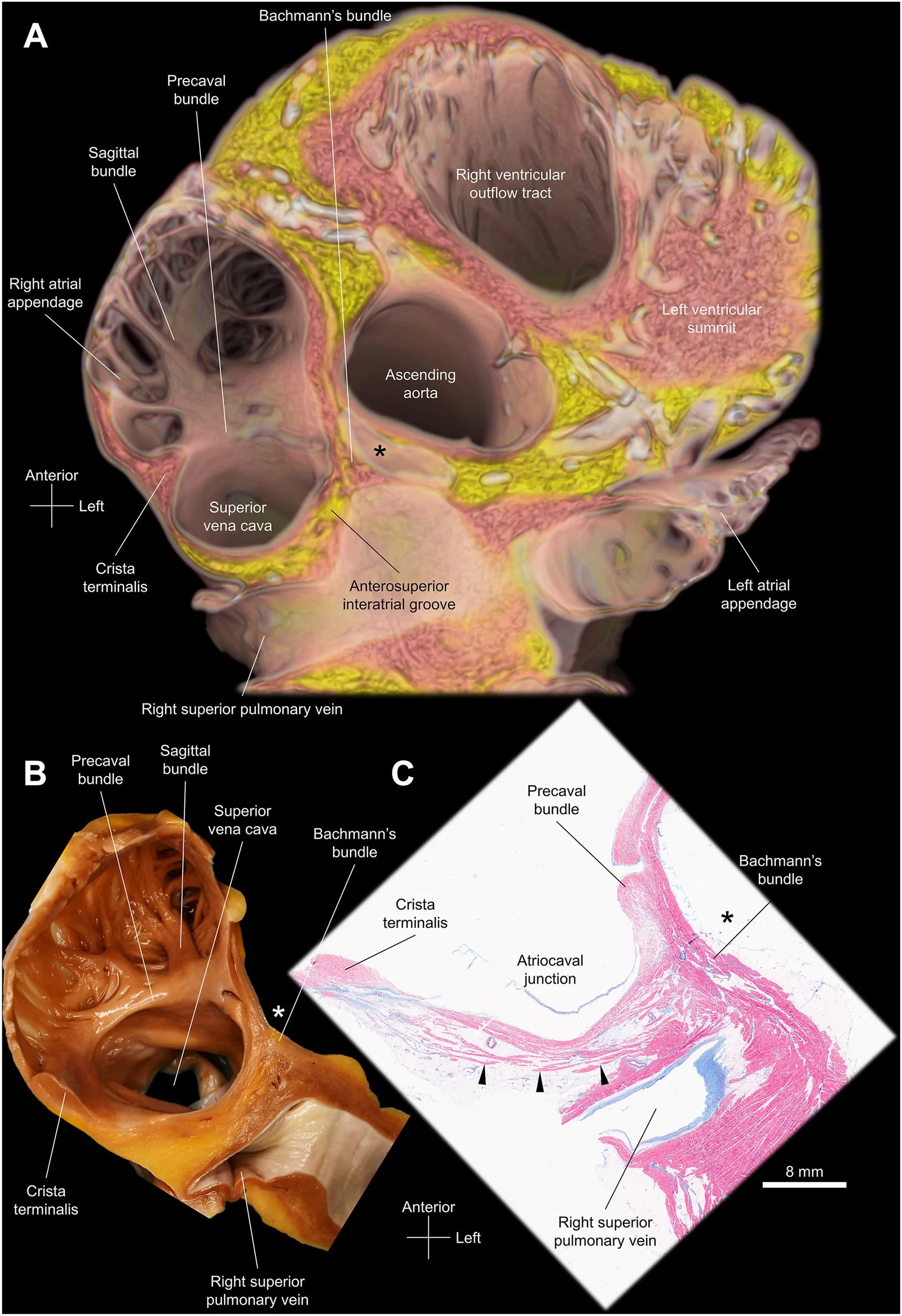
Relevant anatomy of the interatrial portion of Bachmann’s bundle and transverse sinus. The virtual dissection image reconstructed from a pressure-perfused and fixed heart viewed from the inferior direction (**A**), with its corresponding real sample (**B**), highlights the structures surrounding Bachmann’s bundle, including the superior vena cava, precaval bundle, crista terminalis, anterosuperior interatrial groove, ascending aorta, and left atrial appendage. The transverse sinus (*asterisks*) is located between the ascending aorta and Bachmann’s bundle. Identical histology proves Bachmann’s bundle as an epicardial interatrial connection facing the transverse sinus (**C**). The intercaval bundle (*arrowheads*) is also observed as an epicardial myocardial bundle connecting the sinus venarum with the antrum of the right superior pulmonary vein. It takes an oblique course within the interatrial groove.

In 1916, Bachmann demonstrated that interatrial conduction between the right and left atrial appendages could be delayed by crushing the interatrial band of the dog heart.^19^ On average, the interatrial portion of Bachmann’s bundle has 4.59 mm thickness, 6.10 mm height, and 13.8 mm width when measured using cardiac computed tomography.^20^ The bundle continues on the anterior wall of the left atrium and divides to surround the base of the left atrial appendage.^21^ The precaval bundle guarding the anteroinferior orifice of the superior vena cava (Figure 8), which is the superior continuity of the crista terminalis, converges into Bachmann’s bundle at the level of the right atriocaval junction.^21^^,^^22^ Histologically, Bachmann’s bundle is neither composed of any specialized myocardium nor insulated by a fibrous sheath. Preferential conduction via Bachmann’s bundle is likely to be enabled by aggregated cardiomyocytes aligned parallel to Bachmann’s bundle.^15^

Physiological left atrial excitation via Bachmann’s bundle can be observed in the human heart as a breakthrough on the anterior wall, occurring on average 30 milliseconds after the onset of sinus node activation.^23^^,^^24^ Atrial signal sequence observed in coronary sinus EGMs in healthy hearts can exhibit multiple patterns, including 1) collision, 2) simultaneous, or 3) proximal-to-distal activation patterns.^25^ In contrast, patients with interatrial conduction delay can demonstrate an exclusively proximal-to-distal activation pattern.^25^ However, this is not always the case, as observed in the present case. This is because the interpretation of atrial signal sequences observed in coronary sinus EGMs is influenced by the location and number of electrodes placed within the coronary sinus. The efficacy of Bachmann’s bundle pacing, whether endocardial or epicardial, in restoring left atrial activation is likely to vary depending on the relationship between the pacing site and the location and extent of the lesion responsible for the interatrial conduction delay.

### Clinical anatomy of the transverse sinus

**Figure 9:**
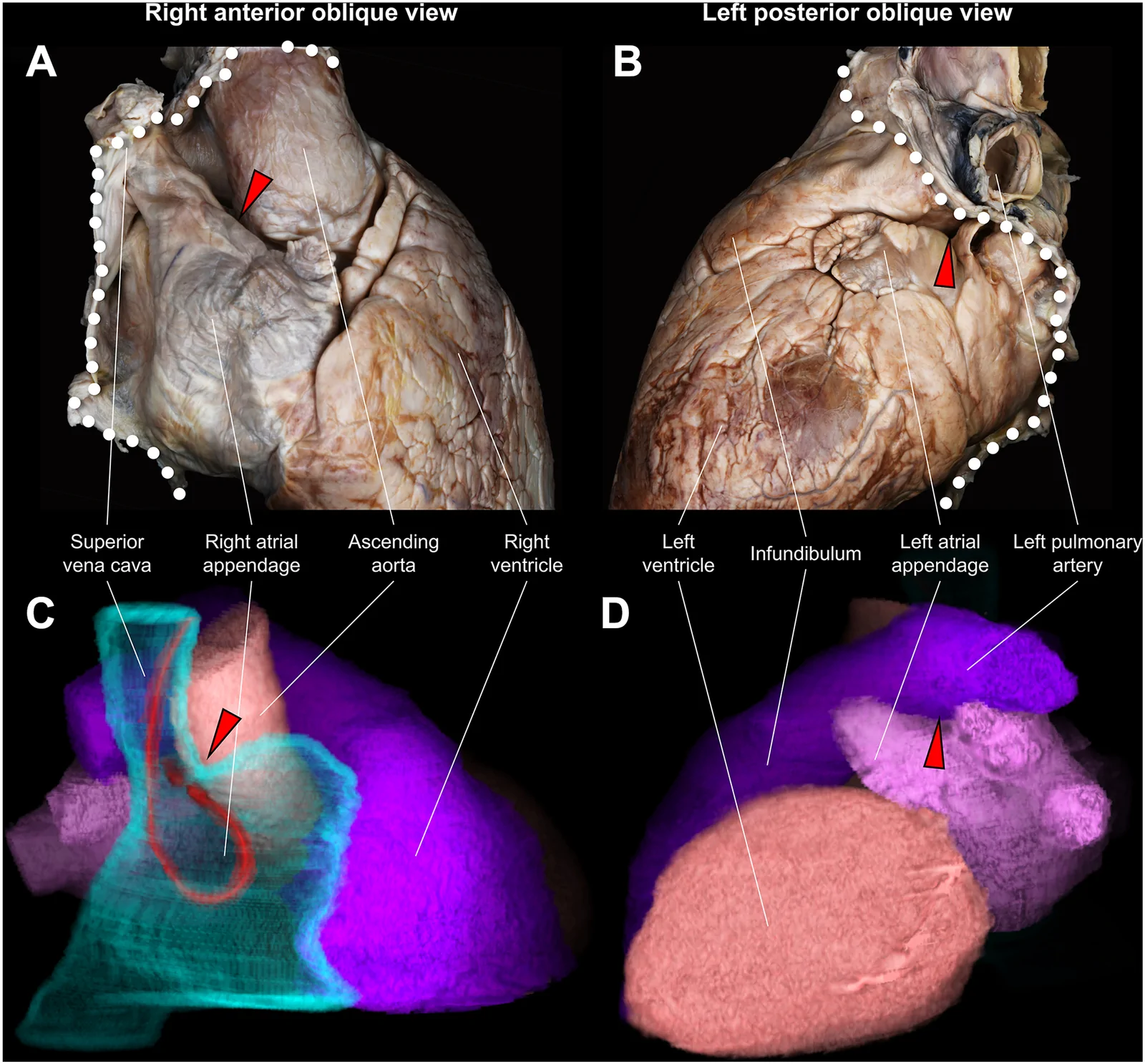
Entrances to the transverse sinus. Pressure-perfused and fixed heart viewed from the right anterior (**A**) and left posterior (**B**) oblique directions denote the right- and left-side entrances (*arrowheads*) to the transverse sinus. The right-side entrance is located between the atriocaval junction and the ascending aorta. The left-side entrance is located between the base of the left atrial appendage and the left pulmonary artery. If you can lift each appendage away from adjacent arterial trunks, it is also feasible to enter the space medially to each appendage. Volume-rendered images reconstructed from the live heart datasets of the present case demonstrate the same anatomy, viewed from the identical directions (**C, D**).

In the dissection suites with an excised heart, or during cardiac surgery under cardiopulmonary support, it is easy to put an index finger into the transverse sinus. However, in physiological settings, the transverse sinus is not a wide-open, straight space; rather, it is a potential space between the ascending aorta/pulmonary root and the anterior wall of the atria, covered by the right pulmonary artery. The right- and left-side entrances to the transverse sinus are guarded by the superior vena cava/right atrial appendage and left atrial appendage/left pulmonary artery, respectively (Figure 9).^26^, ^27^, ^28^

Although the “transverse” sinus sounds as if it is parallel to the right-to-left axis, that is not the case. The transverse sinus extends from the right-anterior to the left-posterior direction. Thus, it does not face the anterior direction, which provides its foreshortened views, but its en-face view can be obtained from the left anterior oblique direction with or without cranial angulation (Figure 6). The right-side entrance to the transverse sinus is located between the superior vena cava and ascending aorta above the aortocaval junction, and the left-side entrance is located at the base of the left atrial appendage beneath the left pulmonary artery (Figure 9). Based on this clinical anatomy, the right anterior oblique view is useful to confirm the retroaortic course of the catheter placed into the transverse sinus (Figure 2). When using the left anterior oblique direction, a catheter moving across the heart does not guarantee transverse sinus insertion. Rather, it is far easier, and potentially safer, to pass anteriorly to the right ventricular outflow tract and pulmonary root because it is a free, open space, not a tunnel-like structure like the transverse sinus.^26^

### Transcatheter epicardial mapping of Bachmann’s bundle

“Bachmann’s bundle pacing” generally refers to the one from the endocardial side targeting the medial area near the right atriocaval junction.^1^^,^^5^^,^^8^^,^^10^, ^11^, ^12^, ^13^^,^^29^ However, as Bachmann’s bundle is essentially an epicardial structure, its selective pacing requires epicardial access from the transverse sinus.^16^^,^^17^ In this regard, the current endocardial approach with an active fixation lead is likely to capture the precaval bundle or its terminal conversion into Bachmann’s bundle in addition to surrounding right atrial myocardium (Figure 8). Thus, it is also reasonable to refer to this endocardial approach as “Bachmann’s bundle area pacing.”^30^ During cardiac surgery, Bachmann’s bundle can easily be visualized as a relatively thick and horizontal muscle strand behind the transverse sinus. Thus, direct pacing is readily feasible and has a favorable outcome in reducing postoperative atrial fibrillation with better pacing threshold as compared to right atrial appendage pacing, especially when reduction of P wave duration is achieved.^16^^,^^17^

Compared to the situation during cardiac surgery, the transcatheter epicardial approach into the transverse sinus is believed to be challenging, given its narrow, tortuous course through the potential space between the arterial trunks and the anterior wall of the atria.^26^ Although there have been multiple reports of transcatheter mapping within the oblique sinus,^31^ to the best of our knowledge, this report is the first-in-human case demonstrating transcatheter epicardial access to the transverse sinus. Of note in this case, an inferior epicardial approach was selected to guide the ablation catheter into the transverse sinus from its left-side entrance around the left atrial appendage. The left-side entrance would be anatomically less challenging when using the inferior epicardial approach, as the entrance opens towards the left lateral direction. Conversely, the anterior epicardial approach would be optimal if the right-side entrance between the superior vena cava and the ascending aorta is selected, as the entrance opens towards the right anterior direction.^22^^,^^27^ In addition, the interatrial portion of Bachmann’s bundle is much closer when using such a right-side approach. In either situation, support of the deflectable sheath would be extremely useful.

**Figure 10:**
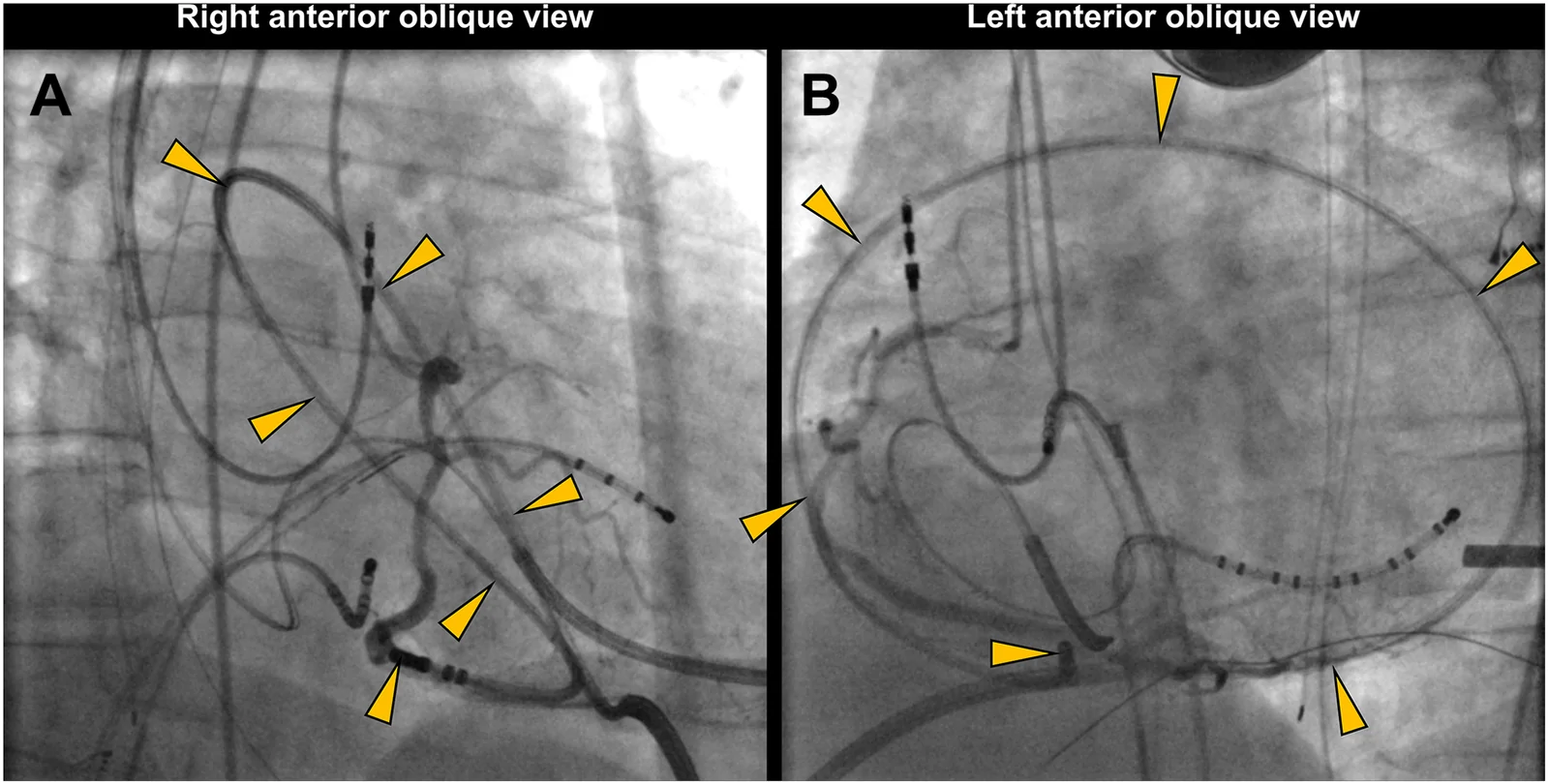
Biplane fluoroscopic image of another case with transcatheter epicardial ablation with an ablation catheter passing through the transverse sinus. An inferior approach is used for epicardial access. Images are viewed from the right (**A**) and left (**B**) anterior oblique directions and captured during the right coronary angiography. The ablation catheter (*arrowheads*) passes through the transverse sinus, entering from the left-side entrance and exiting from the right-side entrance before reaching the basal inferior right ventricle near the distal right coronary artery.

Since this case, we have had multiple cases for whom passage of the catheter through the transverse sinus was successfully achieved (Figure 10). However, as this approach has not been popular, multiple clinical questions remain unresolved at this point. Obviously, just because we can, that does not mean we should.^32^ How feasible is transcatheter access to the transverse sinus across a diverse population with varying cardiac anatomy? What are the potential advantages and risks compared with popular endocardial mapping techniques? How can this transcatheter transverse sinus access be applied to address current challenges, in addition to its application in Bachmann’s bundle pacing?

With such questions in mind, this transverse sinus access is likely to have immense potential to expand the field of cardiac intervention and device implantation. Regular Bachmann’s bundle pacing via the endocardial approach can sometimes be challenging due to a small target area or lipomatous hypertrophy of the atrial septum. Pulmonary vein isolation involving the anterior antrum of the right superior pulmonary vein can create a scar near the target area (Figure 8), which may affect pacing lead performance. Thus, the development of a dedicated epicardial pacing system via the transverse sinus could be an innovative direction in the future of physiological atrial pacing. Such a system can directly capture Bachmann’s bundle with minimized risk of bloodstream infection. Other approaches include epicardial approaches to the left atrial appendage, vestigial fold, left superior pulmonary vein, left atrial roof and anterior wall, aortomitral continuity, anterosuperior interatrial groove, medial free wall of the right atrium, bilateral sinus node artery, superior vena cava, non-coronary aortic sinus, posterolateral attachment of the right ventricle, deep cardiac plexus, and superior right and left atrial ganglionated plexus.^33^, ^34^, ^35^, ^36^, ^37^ At least from the anatomical standpoint, we realize this approach will not always be straightforward,^26^ especially when structures around the transverse sinus exhibit pathological dilatation or increased pressure. Based on a profound knowledge of the correlative anatomy of the transverse sinus and surrounding structures in each case, further clinical experience is necessary to assess the feasibility and safety of its application.

## Conclusion

Transcatheter epicardial Bachmann’s bundle pacing via the transverse sinus is a feasible and useful option to guide subsequent endocardial Bachmann’s bundle pacing in this patient with heart failure, who had a right atrial appendage lead and underwent epicardial ablation via the pericardial space.

## Disclosures

The authors have no conflicts of interest to disclose.
